# Supplementary figures and images for: Unconditioned and learned morphine tolerance influence hippocampal-dependent short-term memory and the subjacent expression of GABA-A receptor alpha subunits
Source: PLoS One. 2021 Sep 9;16(9):e0253902. doi: 10.1371/journal.pone.0253902 (PMC8428970; doi:10.1371/journal.pone.0253902)

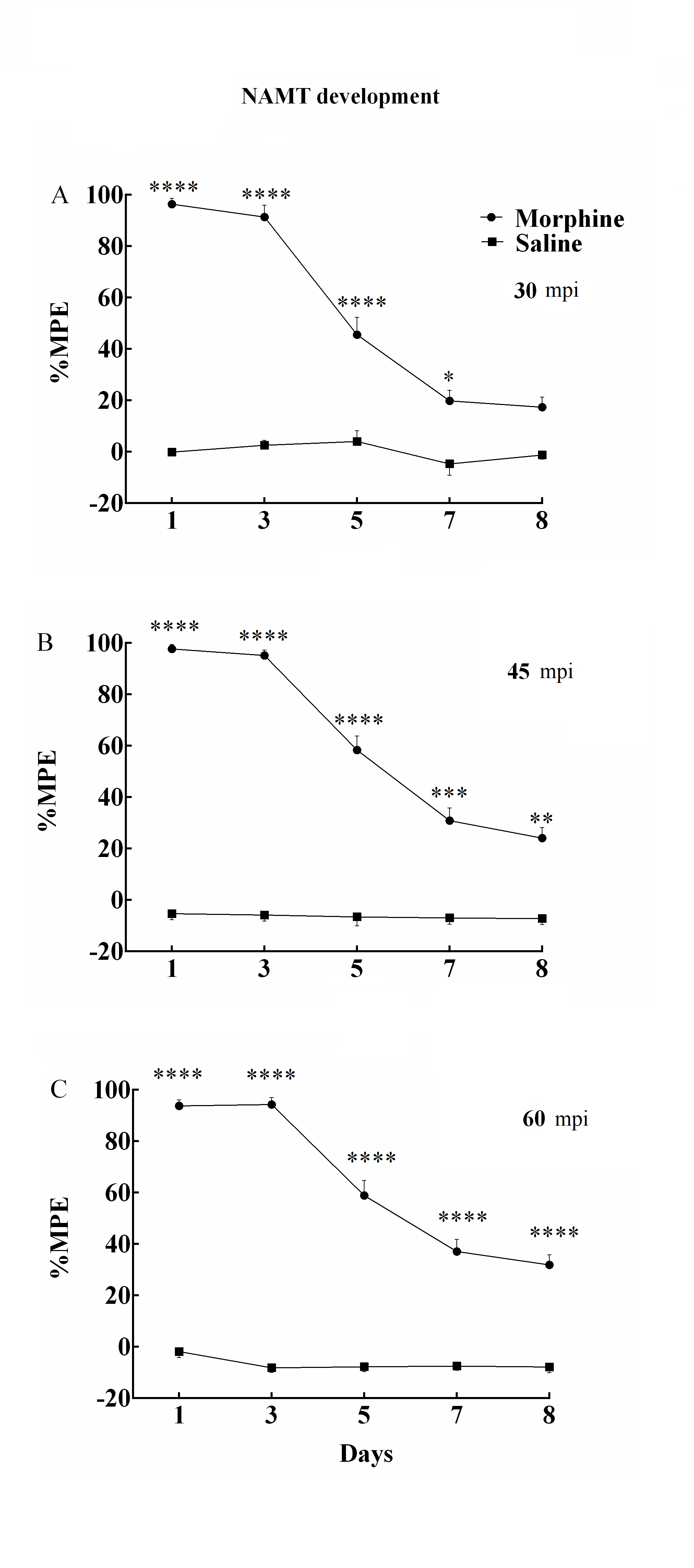

Supplement: S1 Fig — Changes in tail-flick responses were expressed as percentage of maximal possible effect in three time-courses (30, 45, and 60 min past morphine injection. A, B and C, respectively). Values were expressed as mean ± S.E.M, (*P < 0.05, **P < 0.01, ***P < 0.001 and ****P < 0.0001 vs. control (saline injection) group. 1, 3, 5, 7 and 8 represented days post subcutaneous injections (Repeats-measured two-way ANOVA followed by protected Tukey’s test for multiple comparisons), "MPE and NAMT "mean maximal possible effect and non-associative morphine tolerance, respectively. (TIF) [file pone.0253902.s001.tif]

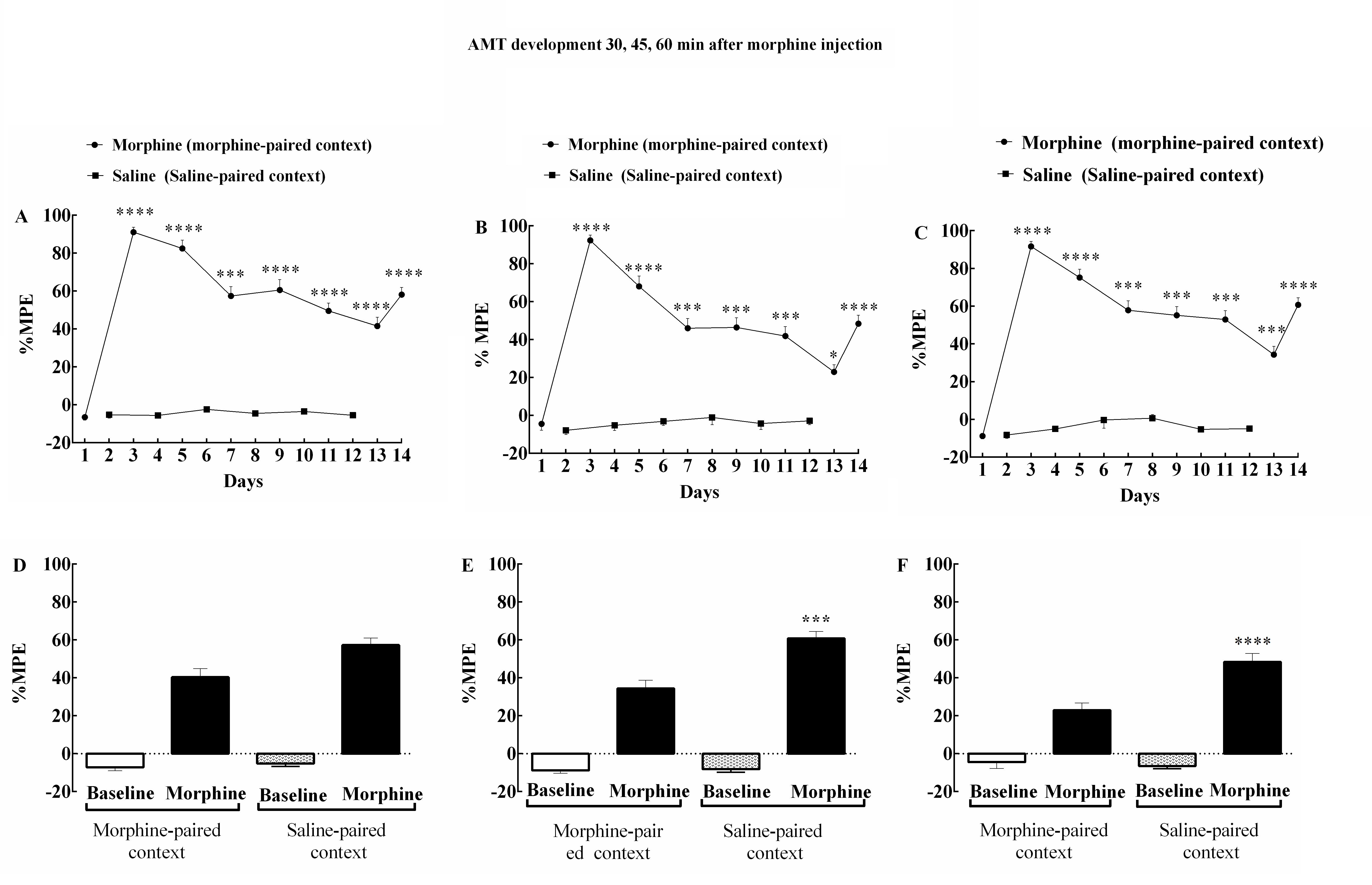

Supplement: S2 Fig — Acquisition of AMT was induced by administration of saline on even days and morphine (4 mg/kg) on odd-days in their distinctive context until day 13. On day 14 morphine was injected in saline-paired environment and %MPE was measured at three time-courses (30, 45, and 60). (D-E-F) AMT is dependent to the injection context. % MPE in morphine-paired and saline-paired context before (baseline) and after (morphine in day 13 and 14) the development of AMT. baseline’s MPE was measured on experimental days 1 and 2, respectively. Animals were tolerant to morphine when tested in the morphine-paired environment on day 13th but day 14th, anti-nociceptive effects of morphine were significantly increased with the same dose of morphine given in the saline-paired environment. Data were expressed as mean±S.E.M, (*P < 0.05, ***P < 0.001 and ****P < 0.0001 vs. control (saline injection) group and baseline. 1–14 represented days post injection (Repeats-measured two-way ANOVA followed by protected Tukey’s test for multiple comparisons). "MPE and AMT" mean maximal possible effect and associative morphine tolerance, respectively. (TIF) [file pone.0253902.s002.tif]

Fig 6-Up

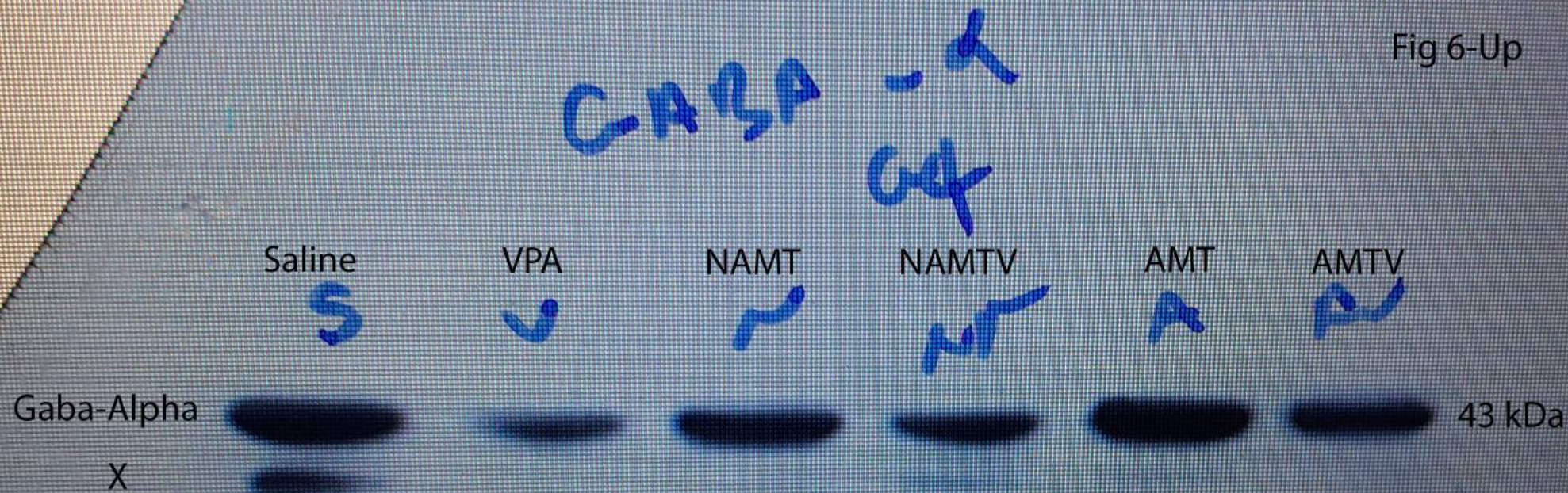

29.11.5

Fig 6-Down

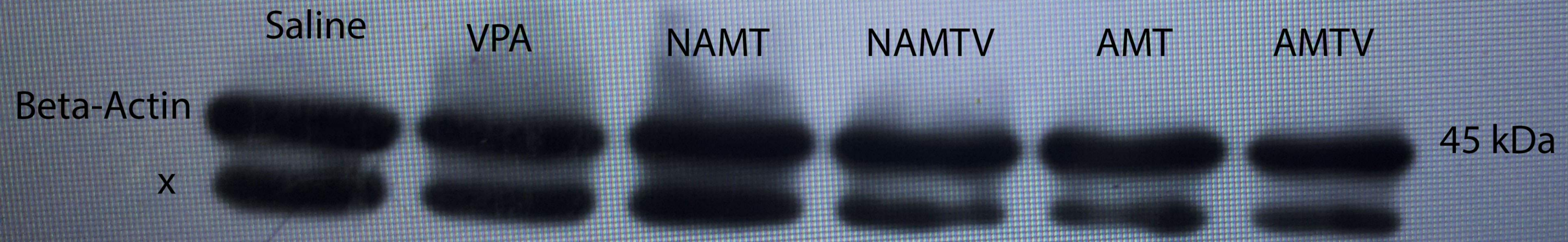

actin

Gg

AMT 0.9

Supplement: S1 Raw images — (PDF) [file pone.0253902.s011.pdf]
