# Supplementary material for: Unconditioned and learned morphine tolerance influence hippocampal-dependent short-term memory and the subjacent expression of GABA-A receptor alpha subunits
Source: PLoS One. 2021 Sep 9;16(9):e0253902. doi: 10.1371/journal.pone.0253902 (PMC8428970; doi:10.1371/journal.pone.0253902)
Supplement: S1 File — Experiment 1. (DOCX) [file pone.0253902.s005.docx]

**Supplementary Fig 1**, Analgesic effect of morphine (4 mg/kg) after consecutive daily injections in NAMT.

| Morphine  1 | Morphine  3 | Morphine  5 | morphine  7 | Morphine  8 |  |
| --- | --- | --- | --- | --- | --- |
| 100 | 100 | 78.76 | 24.44 | 14.44 |  |
| 100 | 100 | 100 | 51.07 | 51.07 |  |
| 100 | 100 | 26.39 | 7.87 | 6.87 |  |
| 100 | 100 | 30.19 | 24.44 | 24.44 |  |
| 100 | 100 | 6.1 | 51.07 | 51.07 |  |
| 100 | 86.21 | 25.39 | 6.87 | 6.87 |  |
| 100 | 100 | 36.15 | 18.45 | 8.45 |  |
| 100 | 100 | 19.91 | 19.37 | 19.37 |  |
| 100 | 100 | 23.85 | 18.45 | 18.45 |  |
| 100 | 100 | 39.04 | 19.37 | 19.37 |  |
| 100 | 100 | 44.79 | -6.65 | -6.65 |  |
| 100 | 100 | 100 | 35.8 | 23.8 |  |
| 72.94 | 29.66 | 100 | 35.52 | 15.52 |  |
| 78.7 | 35.06 | 45.86 | 27.73 | 27.73 |  |
| 100 | 100 | 80.7 | 35.8 | 25.8 |  |
| 100 | 100 | 52.83 | 34.04 | 22.04 |  |
| 100 | 100 | 49.6 | 20 | 18 |  |
| 100 | 100 | 1.11 | 19.37 | 19.37 |  |
| 100 | 100 | 10.32 | 6.68 | 6.68 |  |
| 39.59 | 100 | 27.41 | -6.65 | -5.65 |  |
| 100 | 100 | 19.72 | -8.02 | -7.02 |  |
| 100 | 100 | -4.74 | -13.54 | -13.54 |  |
| 100 | 100 | 100 | 18.45 | 18.45 |  |
| 100 | 100 | 100 | 19.37 | 19.37 |  |
| 100 | -6.49 | -6.73 | -3.76 | -3.76 |  |
| 100 | 100 | 23.72 | 18.57 | 18.57 |  |
| 100 | 96.33 | 100 | -0.08 | -0.08 |  |
| 100 | 100 | 39.33 | 100 | 100 |  |
| 100 | 100 | 100 | 21.49 | 21.49 |  |
| 100 | 100 | -1.77 | 0.34 | 0.34 |  |
|  |  |  |  |  |  |
| 96.37433 | 91.359 | 45.59767 | 19.862 | 17.362 | Avr. |
| 2.25932 | 4.607424 | 6.713845 | 4.091109 | 3.946816 | SEM |

**Experiment 1-A**

**Experiment 1-B**

| Morphine  1 | Morphine  3 | Morphine  5 | Morphine  7 | Morphine  8 |  |
| --- | --- | --- | --- | --- | --- |
| 100 | 100 | 100 | 55.9 | 35.9 |  |
| 100 | 100 | 100 | 55.9 | 45.9 |  |
| 100 | 100 | 59.34 | 34.98 | 21.5 |  |
| 100 | 100 | 79.71 | 55.9 | 65.9 |  |
| 100 | 100 | 59.2 | 52 | 42 |  |
| 100 | 100 | 100 | 8.07 | 8.07 |  |
| 100 | 100 | 100 | 27 | 7 |  |
| 100 | 100 | 70.4 | 25 | 15 |  |
| 100 | 100 | 86.87 | 34 | 27 |  |
| 100 | 100 | 64.04 | 37.24 | 19.24 |  |
| 100 | 100 | 57.24 | 47 | 34 |  |
| 100 | 100 | 100 | 54 | 47 |  |
| 73.01 | 90.77 | 73.1 | 100 | 37 |  |
| 36.39 | 100 | 89.14 | 73.29 | 29.29 |  |
| 100 | 100 | 51.24 | 88.93 | 78.93 |  |
| 100 | 100 | 78.86 | 41.64 | 21.64 |  |
| 100 | 100 | 63.06 | 55.21 | 55.21 |  |
| 100 | 100 | 100 | 64 | 61 |  |
| 100 | 100 | 23.94 | -5.35 | -5.35 |  |
| 100 | 100 | 21.76 | 9.97 | 8.97 |  |
| 100 | 100 | 42.85 | 6.21 | 4.52 |  |
| 98.5 | 100 | 44.64 | 5.73 | 5.73 |  |
| 100 | 100 | 100 | 100 | 100 |  |
| 100 | 100 | 100 | 60.93 | 60.93 |  |
| 100 | 100 | -20.8 | 17.22 | 17.22 |  |
| 100 | 100 | 32.91 | -8.17 | -8.17 |  |
| 100 | 100 | 81.62 | 14.18 | 14.18 |  |
| 100 | 100 | 25.76 | 14.18 | 14.18 |  |
| 100 | 100 | 100 | 46.9 | 36.9 |  |
| 100 | 100 | 52.04 | 46.9 | 46.9 |  |
| 100 | 93.8 | 38.5 | -7.84 | -7.84 |  |
| 100 | 66.03 | 34.89 | 20.76 | 20.76 |  |
| 100 | 100 | 36.69 | 7.1 | 7.1 |  |
| 100 | 100 | 98.57 | 9.7 | 9.7 |  |
| 100 | 72.01 | 20 | 6.36 | 6.36 |  |
| 100 | 100 | 20 | 9 | 9 |  |
| 100 | 30.94 | 7.62 | -3.5 | -3.5 |  |
| 100 | 71.8 | -3.06 | -6.83 | -6.83 |  |
| 100 | 96.17 | 20.98 | -15.86 | -15.86 |  |
| 100 | 84.1 | 23.04 | -1.24 | -1.24 |  |
|  |  |  |  |  |  |
| 97.6975 | 95.1405 | 58.35375 | 30.91025 | 24.131 | Avr. |
| 1.710616 | 2.110482 | 5.382113 | 4.835508 | 4.117029 | SEM |

**Experiment 1-C**

| Morphine 1 | Morphine  3 | Morphine  5 | Morphine  7 | Morphine  8 |  |
| --- | --- | --- | --- | --- | --- |
| 100 | 100 | 100 | 93.46 | 83.46 |  |
| 100 | 100 | 100 | 34 | 24 |  |
| 100 | 100 | 38.76 | 20 | 15 |  |
| 100 | 100 | 37.76 | 21 | 18 |  |
| 100 | 100 | 17.64 | 75.05 | 55.05 |  |
| 100 | 100 | 50.78 | 19.64 | 15.64 |  |
| 100 | 100 | 91.49 | 34 | 33 |  |
| 100 | 100 | 50.64 | 44 | 41 |  |
| 100 | 100 | 88.15 | 96.52 | 76.52 |  |
| 100 | 100 | 69.87 | 54 | 24 |  |
| 44.12 | 100 | 66.04 | 29 | 29 |  |
| 57.22 | 100 | 88.2 | 36 | 36 |  |
| 97.91 | 100 | 100 | 91.4 | 81.4 |  |
| 79.8 | 100 | 100 | 55.46 | 35.46 |  |
| 100 | 100 | 65.25 | 90.01 | 70.01 |  |
| 94.28 | 100 | 100 | 67.4 | 47.4 |  |
| 86.21 | 100 | 78.96 | 44.34 | 44.34 |  |
| 89.91 | 100 | 100 | 24.34 | 24.34 |  |
| 60.6 | 100 | 19.53 | -18.06 | -18.06 |  |
| 84.95 | 48.72 | 18.29 | 40.04 | 40.04 |  |
| 92.91 | 100 | 16.09 | 44.97 | 44.97 |  |
| 100 | 100 | 13.38 | 32.89 | 32.89 |  |
| 100 | 85.07 | 59.74 | 13.92 | 13.92 |  |
| 100 | 37.79 | 14.48 | 9.36 | 9.36 |  |
| 100 | 52.39 | 10.56 | 9.59 | 9.59 |  |
| 100 | 82.49 | 12.85 | 13.55 | 13.55 |  |
| 100 | 99.63 | 91.33 | 34.95 | 34.95 |  |
| 100 | 100 | 100 | 5.69 | 5.69 |  |
| 100 | 99.28 | 24.13 | 8.95 | 8.95 |  |
| 100 | 100 | 11.09 | 25.38 | 25.38 |  |
| 100 | 100 | 63.6 | 27.27 | 27.27 |  |
| 100 | 100 | 28.7 | 36.12 | 36.12 |  |
| 100 | 100 | 100 | 36.07 | 36.07 |  |
| 100 | 100 | 75.19 | 10.39 | 10.39 |  |
|  |  |  |  |  |  |
| 93.76206 | 94.27559 | 58.89706 | 37.07941 | 31.90294 | Avr. |
| 2.34994 | 2.700507 | 5.886178 | 4.732007 | 3.859691 | SEM |
