# Supplementary material for: Unconditioned and learned morphine tolerance influence hippocampal-dependent short-term memory and the subjacent expression of GABA-A receptor alpha subunits
Source: PLoS One. 2021 Sep 9;16(9):e0253902. doi: 10.1371/journal.pone.0253902 (PMC8428970; doi:10.1371/journal.pone.0253902)
Supplement: S2 File — Experiment 2. (DOCX) [file pone.0253902.s006.docx]

**Supplementary Fig 2,** Analgesic effect of morphine (4 mg/kg) after consecutive daily injections in AMT.

**Experiment 2-A**

| Morphine 1 | Morphine 3 | Morphine 5 | Morphine 7 | Morphine 9 | Morphine 11 | Morphine 13 | Morphine 14 | |
| --- | --- | --- | --- | --- | --- | --- | --- | --- |
| -10.54 | 100 | 60.84 | 37.02 | 31.64 | 25.65 | -0.84 | 63.97 | |
| -4.32 | 48.45 | 96.46 | 18.84 | 28.66 | 16.19 | 11.35 | 65.14 | |
| 3.33 | 100 | 100 | 100 | 100 | 100 | 35.13 | 9.95 | |
| 6.6 | 100 | 90.32 | 21.12 | 99.8 | 37.48 | 39.12 | 32.02 | |
| -13.3 | 38.81 | 100 | 63.6 | 21.93 | 52.37 | 87.55 | 54.29 | |
| -12.85 | 75.8 | 100 | 100 | 100 | 94.98 | 61.91 | 41.78 | |
| 13.03 | 100 | 100 | 2.07 | 100 | 95.79 | 25.08 | 72.9 | |
| -10.11 | 100 | 100 | -8.58 | 100 | 92.94 | 38.46 | 90.61 | |
| -6.86 | 100 | 8.99 | 37.5 | 61.92 | 84.05 | 19.53 | 54.08 | |
| 2.3 | 48.06 | 8.79 | 75.5 | 100 | 27.17 | 97.06 | 60.06 | |
| -9.93 | 77.46 | 100 | 80.5 | 44.79 | 37.85 | 13.75 | 85.87 | |
| -18.34 | 100 | 77.06 | 46.1 | 100 | 35.83 | 24.68 | 33.09 | |
| -0.32 | 100 | 93.34 | 84.9 | 82.68 | 62.2 | 15.63 | 51.79 | |
| -6.79 | 94.02 | 100 | 45.15 | 100 | 31.44 | 25.51 | 28.74 | |
| -8.86 | 100 | 100 | 55.81 | 4.79 | 35.57 | 15.47 | 31.8 | |
| -8.15 | 100 | 59.31 | 26.2 | 18.78 | 17.71 | 8.31 | 39.96 | |
| -6.99 | 100 | 100 | 20.9 | 34.98 | 58.53 | 68.84 | 100 | |
| -15.32 | 85.88 | 27.64 | 68.99 | 4.31 | 30.11 | 100 | 70.84 | |
| -0.92 | 100 | 100 | 21.34 | 48.48 | 21.45 | 83.98 | 100 | |
| -8.35 | 100 | 14.46 | 25.84 | 64.86 | -2.12 | 36.84 | 30.33 | |
| 0.83 | 100 | 100 | 78.3 | 68.56 | 46.71 | 17.47 | 89.23 | |
| -9.05 | 100 | 30.86 | 100 | 92.12 | 16.64 | 9.9 | 73.86 | |
| -1.84 | 28.47 | 34.53 | -3.33 | 9.07 | 31.07 | 3.56 | 19.59 | |
| -2.17 | 73.34 | 17.87 | 55.46 | 61.16 | 29.27 | 2.82 | 37.32 | |
| 2.77 | 100 | 100 | 84.9 | 82.68 | 62.2 | 15.63 | 51.79 | |
| -5.69 | 94.02 | 100 | 45.15 | 100 | 31.44 | 25.51 | 28.74 | |
| -8.86 | 100 | 100 | 55.81 | 4.79 | 35.57 | 15.47 | 31.8 | |
| -8.15 | 100 | 59.31 | 26.2 | 18.78 | 17.71 | 8.31 | 39.96 | |
| -6.99 | 100 | 100 | 20.9 | 34.98 | 58.53 | 68.84 | 100 | |
| -15.32 | 85.88 | 100 | 68.99 | 4.31 | 30.11 | 100 | 70.84 | |
| -17.65 | 100 | 100 | 27.19 | 100 | 76.27 | 100 | 95.66 | |
| -15.32 | 100 | 100 | 86.79 | 100 | 96.17 | 72.4 | 40.8 | |
| -18.49 | 100 | 100 | 0 | 0 | 17 | 100 | 100 | |
| 6.96 | 100 | 100 | 100 | 100 | 22.67 | 72.16 | 71.25 | |
| 13.1 | 100 | 100 | 92.73 | 81.69 | 76.19 | 100 | 100 | |
| 8.61 | 79.38 | 71.8 | 82.24 | 67.72 | 100 | 27.77 | 100 | |
| -8.15 | 100 | 100 | 68.26 | 19.59 | 59.2 | 35.87 | 22.64 | |
| 6.96 | 100 | 100 | 75.64 | 34.23 | 50.8 | 17.48 | 26.58 | |
| -1.84 | 88.64 | 100 | 72.92 | 22.86 | 21.79 | 35.55 | 39.19 | |
| -1.84 | 100 | 100 | 7.08 | 86.23 | 31.32 | 43.58 | 51.6 | |
| -1.84 | 100 | 100 | 94.51 | 58.66 | 24.4 | 21.31 | 59.4 | |
| -1.84 | 64.26 | 27.23 | 23.46 | 41.91 | 26.68 | 53.62 | 68.53 | |
| -40.97 | 100 | 100 | 100 | 100 | 100 | 76.03 | 32.83 | |
| 17.41 | 100 | 90.26 | 100 | 100 | 60.58 | 25.4 | 76.14 | |
| -23.9 | 90.5 | 97.38 | 69.95 | 100 | 65.71 | 79.28 | 22.39 | |
| -18.63 | 100 | 100 | 100 | 99.53 | 72.79 | 23.88 | 88.96 | |
| -14.5 | 100 | 93.39 | 100 | -25.69 | 79.68 | 9.81 | 69.8 | |
| -27.92 | 100 | 100 | 100 | 97.62 | 83.22 | 26.78 | 65.71 | |
|  |  |  |  |  |  |  |  | |
| -6.47938 | 91.10354 | 82.49667 | 57.41563 | 60.59208 | 49.56063 | 41.57896 | 58.16313 | Avr. |
| 1.572065 | 2.545109 | 4.315739 | 4.908687 | 5.506957 | 4.097972 | 4.623932 | 3.8068 | SEM. |

| Saline 2 | Saline 4 | Saline 6 | Saline 8 | Saline 10 | Saline 12 |  |
| --- | --- | --- | --- | --- | --- | --- |
| -3.27 | -11.4 | -2.86 | 4.47 | -1.81 | -2.37 |  |
| -3.74 | 5.28 | -14.01 | 1.78 | -6.8 | 0.08 |  |
| 0.82 | 0.82 | -6.56 | -10.28 | -5.59 | -4.01 |  |
| -21.9 | -3.9 | -9.11 | -11.2 | -14.03 | -7.7 |  |
| 2.46 | -5.63 | -16.03 | 0.07 | -21.83 | -9.72 |  |
| 1.5 | 0.87 | -3.6 | -12.6 | -13.51 | -1.23 |  |
| -9.85 | -5.16 | 18.06 | -1.91 | 2.01 | 1.23 |  |
| -1.19 | -5.14 | -5.76 | -5.48 | -5.51 | -6.15 |  |
| -8.27 | -14.55 | -1.61 | -14.29 | -6.77 | -7.26 |  |
| -9.43 | -19.61 | -23.98 | -20.05 | 1.32 | -14.9 |  |
| -7.05 | 1.34 | 0.37 | -8.91 | -5.79 | -26.25 |  |
| -16.03 | -5.17 | 10.51 | -10.43 | -9.27 | -15.83 |  |
| 6.5 | -6.25 | -9.29 | -3.71 | -19.75 | -11.58 |  |
| -8.13 | -17.19 | 6.43 | -8.3 | 3.34 | 2.73 |  |
| -4.06 | -6.96 | 6.56 | -30.39 | -5.75 | -8.23 |  |
| -9.85 | -5.16 | 18.06 | -1.91 | 2.01 | 1.23 |  |
| -1.19 | -5.14 | -5.76 | -5.48 | -5.51 | -6.15 |  |
| -8.27 | -14.55 | -1.61 | -14.29 | -6.77 | -7.26 |  |
| -3.87 | -0.06 | 3.57 | -9.93 | -3.65 | -10 |  |
| 1.55 | -39.43 | -0.46 | -0.71 | -31.58 | -17.11 |  |
| -11.33 | -18.57 | -24.99 | 9.04 | 13.07 | -9.47 |  |
| -9.43 | -19.61 | -23.98 | -20.05 | 1.32 | -14.9 |  |
| -7.05 | 1.34 | 0.37 | -8.91 | -5.79 | -26.25 |  |
| -16.03 | -5.17 | 10.51 | -10.43 | -9.27 | -15.83 |  |
| 33.19 | -11.67 | 5.38 | -17.8 | 9.520001 | 12.65 |  |
| 6.52 | 11.83 | 2.64 | 1.13 | -5.79 | 8.57 |  |
| -12.92 | -13.78 | -10.61 | -4.06 | -21.17 | 0.42 |  |
| -5.42 | -9.01 | -14.92 | -4.62 | -6.8 | 0.88 |  |
| -17.77 | -7.88 | 7.44 | -10.49 | -9.67 | 0.67 |  |
| -4.3 | -7.63 | -18.27 | -16.59 | 2.41 | 9.6 |  |
| 3.13 | -3.18 | 0.95 | -6.52 | 2.41 | -4.02 |  |
| 2.66 | 3.87 | -7.95 | 4.89 | 2.41 | 5.69 |  |
| 5.44 | -11.42 | -0.5 | 1.37 | 2.41 | -9.47 |  |
| -2.54 | -6.42 | 2.1 | 1.52 | 2.41 | -15.8 |  |
| -1.24 | -1.52 | 4.74 | 9.55 | 2.41 | -2.67 |  |
| -0.41 | 2.79 | 2.16 | -1.21 | 2.41 | -0.34 |  |
| -22.13 | 2.03 | -0.23 | -0.46 | -13.47 | -8.72 |  |
| -48.35 | -8.38 | -0.42 | -3.95 | 5.63 | 3.1 |  |
| -6.32 | -5.37 | -2.57 | -7.11 | -15.48 | 3.62 |  |
| 2.3 | -5.68 | -2.43 | -12.46 | -8.75 | 3.62 |  |
| -18.02 | -5.4 | -0.15 | -0.89 | 15.47 | -14.1 |  |
| -17.09 | -4.07 | -6.28 | 3.79 | -8.65 | -7.98 |  |
| 6.97 | 3.46 | -4.39 | 9.53 | -23.79 | 3.79 |  |
| -5.57 | -2.47 | -0.62 | 5.65 | 6.49 | 17.74 |  |
| -13.46 | 3.62 | 2.08 | 3.62 | 3.62 | 3.62 |  |
| 16.35 | 10.81 | -3.14 | 1.98 | 25.02 | -1.46 |  |
| 0.76 | -9.1 | 11.59 | 10.62 | 6.93 | -27.77 |  |
| -7.02 | 7.69 | -3.53 | 8.98 | 13.01 | -27.96 |  |
|  |  |  |  |  |  |  |
| -5.25729 | -5.53917 | -2.33542 | -4.52979 | -3.4775 | -5.48438 | Avr. |
| 1.681501 | 1.283613 | 1.385534 | 1.288542 | 1.552206 | 1.477681 | SEM |

**Experiment 2-B**

| Morphine  1 | Morphine  3 | Morphine 5 | Morphine  7 | Morphine  9 | Morphine 11 | Morphine  13 | Morphine  14 |  |
| --- | --- | --- | --- | --- | --- | --- | --- | --- |
| -4.84 | 100 | 99.15 | 30.13 | 38.43 | 31 | 19.92 | 53.59 |  |
| -8.89 | 85.08 | 100 | 44.91 | 27.58 | 21.95 | -1.4 | 50.9 |  |
| -6.72 | 100 | 67.35 | 4.6 | 21.72 | 58.92 | 100 | 95.68 |  |
| -8.38 | 100 | 99.04 | 18.08 | 32.85 | 25.49 | 20.81 | 34.14 |  |
| -3.62 | 100 | 45.08 | 76.47 | 100 | 98.05 | -6.47 | 77.31 |  |
| 7.58 | 100 | 0.97 | 68.14 | 68.96 | 30.66 | 32.07 | 21.03 |  |
| -0.06 | 100 | 100 | 89.93 | 100 | 53.01 | 17.88 | 84.42 |  |
| 1.22 | 100 | 38.48 | 100 | 100 | 48 | 10.34 | 100 |  |
| -1.65 | 8 | 41.74 | 34.27 | -4.57 | 47 | 1.94 | 11.34 |  |
| 4.4 | 100 | 8.19 | 48.07 | 60.44 | 7.07 | 4.2 | 67.22 |  |
| -0.15 | 100 | 88.85 | 72.9 | 100 | 81.77 | 29.88 | 58.84 |  |
| -5.91 | 73.39 | 100 | 43.45 | 11.99 | 25.21 | 13.5 | 40.57 |  |
| -4.84 | 100 | 99.15 | 30.13 | 38.43 | 31 | 28.57 | 53.59 |  |
| -8.89 | 85.08 | 100 | 44.91 | 27.58 | 21.95 | -1.77 | 51.1 |  |
| -6.72 | 100 | 67.35 | 4.6 | 21.72 | 58.92 | 100 | 95.68 |  |
| -8.38 | 100 | 99.04 | 18.08 | 32.85 | 25.49 | 24.31 | 34.14 |  |
| -14.67 | 100 | 100 | 100 | 100 | 100 | 100 | 91.15 |  |
| -11.95 | 100 | 100 | 100 | 56 | 100 | 100 | 77.04 |  |
| -15.13 | 56.16 | 100 | 100 | 46 | 100 | 100 | 100 |  |
| -16.2 | 100 | 100 | 100 | 100 | 100 | 52.7 | 90.5 |  |
| -7.31 | 100 | 100 | 83.14 | 100 | 100 | 68.97 | 82.11 |  |
| -1.27 | 100 | 72.86 | 100 | 65.41 | 86.58 | 38.95 | 100 |  |
| -3.81 | 100 | 87.78 | 35.13 | 32.76 | 50.69 | 32.19 | 41.54 |  |
| -4.24 | 100 | 14.75 | 95.95 | 11.89 | 69.82 | 11.48 | 26.13 |  |
| -7.85 | 100 | 100 | 63.31 | 33.51 | 24.09 | 100 | 48.96 |  |
| -6.78 | 89.45 | 100 | 47.46 | 74.15 | 45.25 | 14.01 | 56.86 |  |
| -9.22 | 100 | 100 | 100 | 21.4 | 40.87 | 27.08 | 69.99 |  |
| -13.66 | 71.56 | 23.03 | 24.44 | 8.55 | 47.39 | 23.81 | 71.29 |  |
| -4.1 | 87.85 | 21.95 | 66.74 | 46.46 | 78.11 | 67.09 | 95.29 |  |
| -20.22 | 100 | 32.3 | 17.48 | 37.06 | 52.97 | 40.32 | 100 |  |
| -21.4 | 100 | 31.07 | 15.12 | 39.59 | 36.62 | 13.59 | 38.9 |  |
| -1.81 | 100 | 92.54 | -7.28 | 3.82 | 10.6 | 11.69 | 29.9 |  |
| -19.28 | 100 | 50.57 | 11.21 | 57.11 | 4.15 | 4.04 | 56.36 |  |
| -38.51 | 100 | 60.27 | 30.92 | 64.49 | 7.67 | 2.71 | 39.45 |  |
| -17.28 | 100 | 23.37 | 12.28 | 29.75 | 8.61 | -7.8 | 22.54 |  |
| -6.6 | 100 | 100 | 100 | 27.35 | 59.71 | 5.42 | 37.77 |  |
| -48.54 | 100 | 100 | 100 | 70.46 | 94.72 | 94.24 | 45.43 |  |
| -9.89 | 100 | 100 | 100 | 46.89 | 58.61 | 36.23 | 95.15 |  |
| -30.64 | 100 | 100 | 100 | 100 | 100 | 43.68 | 42.2 |  |
| -16.83 | 100 | 100 | 100 | 100 | 100 | 6.3 | 100 |  |
| -16.59 | 100 | 100 | 100 | 73.17 | 100 | -0.24 | 72.61 |  |
| -13.16 | 100 | 100 | 100 | 100 | 100 | 32.02 | 100 |  |
|  |  |  |  |  |  |  |  |  |
| -10.2279 | 93.8986 | 75.92744 | 59.73279 | 51.99512 | 55.05023 | 33.15721 | 62.8207 | Avr. |
| 1.602874 | 2.508523 | 5.004247 | 5.508585 | 4.97087 | 4.973191 | 5.218809 | 4.099848 | SEM |

| Saline 2 | Saline 4 | Saline 6 | Saline 8 | Saline 10 | Saline 12 |  |
| --- | --- | --- | --- | --- | --- | --- |
| -20.3 | -5.99 | 0.77 | 7.29 | -9.39 | -9.91 |  |
| 7.65 | -24.77 | 0.21 | -0.39 | 0.9 | -3.73 |  |
| -17.63 | -21.66 | -21.53 | -5.06 | -7.41 | 1.79 |  |
| -29.53 | -9.98 | -8.54 | -16.41 | -2.35 | -15.28 |  |
| -12.11 | -15.62 | -0.89 | -17.62 | -4.02 | -7.63 |  |
| 3.11 | -5.02 | -4.08 | -13.36 | -13.87 | 1.74 |  |
| -12.34 | -3.77 | 0.22 | 2.73 | -4.6 | 6.86 |  |
| -5.3 | 2.26 | 5.77 | -4.62 | -3.39 | 4.28 |  |
| -17.85 | -8.95 | -0.25 | 6.98 | -11.04 | -5.6 |  |
| -10.84 | -22.06 | -16.65 | 6.64 | -9 | -25.49 |  |
| -0.73 | -19 | -12.32 | -2.57 | -35.05 | -2.11 |  |
| -12.79 | 4.28 | -12.22 | -5.72 | -4.56 | -6.4 |  |
| 3.7 | -12.53 | -13.59 | 5.5 | 2.27 | -14.69 |  |
| -4.93 | -0.07 | -1.01 | 10.59 | 4.92 | -22.37 |  |
| -16.38 | 1 | -29.88 | 3.67 | -10.23 | -6.01 |  |
| -12.34 | -3.77 | 0.22 | 2.73 | -4.6 | 6.86 |  |
| -5.3 | 2.26 | 5.77 | -4.62 | -3.39 | 4.28 |  |
| -17.85 | -8.95 | -0.25 | 6.98 | -11.04 | -5.6 |  |
| -0.13 | 0.49 | -7.05 | 0.71 | -4.17 | 2.88 |  |
| 0.39 | 1.85 | 11.32 | -32.21 | -12.05 | -39.3 |  |
| -5.34 | -22.83 | -3.47 | 19.28 | -2.8 | -24.47 |  |
| -10.84 | -22.06 | -16.65 | 6.64 | -9 | -25.49 |  |
| -0.73 | -19 | -12.32 | -2.57 | -35.05 | -2.11 |  |
| -12.79 | 4.28 | -12.22 | -5.72 | -4.56 | -6.4 |  |
| 47.35 | 3.1 | -1.92 | 9.75 | 16.28 | 1.95 |  |
| -18.23 | -3.42 | -10.66 | 1.22 | 9.08 | 9.37 |  |
| -16.28 | -7.03 | -1.89 | -14.14 | 1.62 | -7.88 |  |
| -13.27 | -12.46 | -5.37 | -3.07 | -3.88 | -10.56 |  |
| -20.52 | 12.08 | -12.43 | 3.1 | -8.32 | -10.01 |  |
| -4.02 | -8.06 | -7.65 | 0.69 | -0.52 | -6.71 |  |
| -0.06 | 2.14 | -4.7 | -3.04 | 1.77 | -0.19 |  |
| 0.06 | 3.32 | 8.21 | -10.01 | 1.58 | 6.09 |  |
| 1.65 | 11.75 | 3.47 | -10.71 | 3.07 | -9.38 |  |
| 3.23 | -1.64 | -0.63 | -7.53 | 0.28 | -11.33 |  |
| -2.84 | -3.87 | 2.11 | 8.13 | 6.74 | 2 |  |
| 1.55 | -6.17 | -3.18 | -5.27 | 3.79 | 5.58 |  |
| -34.09 | -5.03 | 0.11 | -1.59 | -6.34 | -10.36 |  |
| -57.86 | -1.12 | -2.93 | 15.51 | 0.64 | -1.72 |  |
| -9.55 | 4.47 | -7.04 | 48.13 | -0.66 | 3.06 |  |
| -4.42 | -2.7 | -12.72 | 8.26 | -7.43 | 0.78 |  |
| -17.64 | -5.92 | 5.34 | 35.5 | 7.55 | -2.99 |  |
| -15.8 | -3.33 | -8.1 | -7.72 | 4.42 | -6.55 |  |
| 1.88 | -4.77 | -15.65 | 4.58 | 6.61 | 15.15 |  |
| 4.65 | -0.28 | -2.08 | -4.42 | -1.1 | 8.89 |  |
| -7.76 | 6.94 | -8.62 | -0.26 | -24.45 | -5.33 |  |
| -7.94489 | -5.36213 | -0.15617 | 0.66234 | -4.92723 | -5.32872 | Avr. |
| 2.095303 | 1.359069 | 5.113277 | 1.849792 | 1.57387 | 1.594423 | SEM |

**Experiment 2-C**

| Morphine  1 | Morphine  3 | Morphine 5 | Morphine  7 | Morphine  9 | Morphine 11 | Morphine  13 | Morphine  14 |  |
| --- | --- | --- | --- | --- | --- | --- | --- | --- |
| 3.53 | 100 | 40.02 | 8.9 | 16.07 | 81.98 | -4.85 | 46.26 |  |
| -4.09 | 48.45 | 29.22 | -11.12 | 24.5 | -8.55 | -2.64 | 19.97 |  |
| -9.05 | 100 | 100 | 100 | 17.54 | 100 | 8.38 | 83.49 |  |
| 8.16 | 100 | 61.17 | -2.01 | 36.84 | 0.91 | 40.75 | 44.99 |  |
| -14.56 | 85.67 | 100 | 10.61 | 32.48 | 39.3 | 17.35 | 42.61 |  |
| -8.31 | 5.28 | 100 | 3.52 | 4.03 | 48.36 | 38.75 | 36.98 |  |
| 12.03 | 100 | 100 | 5.41 | 100 | 100 | -10.32 | 56.38 |  |
| 5.22 | 100 | 100 | -2.54 | 100 | 100 | 11.22 | 65.29 |  |
| -9.15 | 100 | -4.62 | 37.76 | 100 | 100 | 20.58 | 12.3 |  |
| 3.68 | 100 | 18.9 | 21.22 | 85.83 | 7.16 | 23.9 | 59.4 |  |
| -9.51 | 100 | 100 | 31.04 | 51.29 | 36.46 | 30.83 | 49.46 |  |
| -12.4 | 100 | 70.14 | 79.61 | 63.77 | 27.93 | 9.95 | 22.15 |  |
| -9.21 | 100 | 100 | 79.37 | 46.1 | 79.42 | 14.2 | 59.76 |  |
| -6.19 | 100 | 99.07 | 51.09 | 32.52 | 25.1 | 19.1 | 56.43 |  |
| -10.65 | 100 | -42.05 | 27.98 | 5.8 | 47.76 | 12.85 | 8.41 |  |
| -8.96 | 100 | 100 | 30.8 | 27.39 | 19.42 | 7.87 | 27.12 |  |
| -5.91 | 100 | 70.09 | 4.57 | 34.52 | 8.98 | 88.26 | 100 |  |
| -0.87 | 86 | 90.09 | 100 | 0.2 | 21.5 | 13.79 | 21.31 |  |
| 1.84 | 100 | 100 | 45.16 | 100 | 83.75 | 22.65 | 100 |  |
| 4.63 | 100 | 37.67 | 100 | 40.13 | 4.03 | -3.58 | 2.39 |  |
| -1.22 | 100 | 70.2 | 100 | 51.52 | -1.73 | 12.55 | 94.94 |  |
| -7.29 | 100 | 12.47 | 100 | 100 | 91.08 | 51.88 | 100 |  |
| 1.4 | 10.43 | 16.99 | -6.93 | 13.23 | 3.79 | 9.42 | 6.42 |  |
| 2.1 | 24.38 | 6.71 | 27.96 | 10.67 | 11.77 | -0.92 | 29.05 |  |
| -5.85 | 100 | 84.12 | 79.37 | 46.1 | 79.42 | 14.2 | 59.76 |  |
| -5.1 | 100 | 82.96 | 51.09 | 32.52 | 25.1 | 19.1 | 56.43 |  |
| -10.65 | 100 | 100 | 27.98 | 5.8 | 47.76 | 12.78 | 8.41 |  |
| -8.96 | 100 | 100 | 30.8 | 27.58 | 19.42 | 7.87 | 27.12 |  |
| -5.91 | 100 | 70.09 | 4.57 | 34.52 | 8.98 | 88.26 | 100 |  |
| -0.87 | 86 | 90.09 | 100 | 0.2 | 21.5 | 13.79 | 21.31 |  |
| -4.81 | 100 | 100 | 100 | 94.07 | 100 | 70.7 | 100 |  |
| -8.75 | 100 | 100 | 79.18 | 100 | 100 | 65.55 | 42.99 |  |
| -15.04 | 100 | 100 | 100 | 100 | 100 | 76.69 | 67.92 |  |
| -8.1 | 80.32 | 100 | 61.19 | 100 | 100 | 100 | 100 |  |
| -2.25 | 100 | -0.35 | 100 | 28.88 | 100 | 47 | 100 |  |
| 105.77 | 100 | 100 | 43.47 | -2.04 | 17.52 | 29.53 | 12.24 |  |
| -3.91 | 100 | 37.14 | 30.92 | 16.89 | 66.56 | 15.28 | 44.25 |  |
| 107.63 | 100 | 100 | 56.69 | 62.98 | 14.61 | 29.36 | 28.02 |  |
| -19.82 | 100 | 62.38 | 86.03 | 100 | 32.72 | 37.08 | 28.41 |  |
| -25.72 | 100 | 100 | 20.49 | 27.53 | 32.06 | 5.79 | 36.43 |  |
| 13.43 | 97.24 | 100 | 52.94 | 69.17 | 7.28 | 14.15 | 54.49 |  |
| -0.43 | 99 | 6.49 | 43.32 | 19.7 | 65.75 | 4.18 | 36.12 |  |
| -19.85 | 100 | 19.77 | -7.95 | -3.42 | 27.9 | 7.81 | 100 |  |
| -29.41 | 79.7 | 31.24 | 26.2 | -1.06 | -15.98 | 13.46 | 17.61 |  |
| -16.61 | 100 | 100 | 2.27 | 6.05 | 9.2 | 12.39 | 11.36 |  |
| -17.52 | 100 | 6.31 | 3.93 | 28.34 | 12.25 | 0.96 | -6.66 |  |
| -32.63 | 89.55 | 17.37 | 27.1 | 31.82 | 14.08 | 2.39 | -8.49 |  |
| -19.18 | 82.14 | -20.02 | 7.82 | 31.14 | 0.41 | -10.47 | 25.99 |  |
| -3.09 | 100 | 48.94 | 22.58 | 11.19 | 9.78 | 3.32 | 20.74 |  |
| -50.05 | 100 | 100 | 37.3 | 100 | 100 | 100 | 71.34 |  |
| -8.14 | 100 | 100 | 54.6 | 100 | 55.7 | 35.47 | 60.54 |  |
| -20.4 | 100 | 100 | 100 | 100 | 49.58 | 12.06 | 45.78 |  |
| -23.49 | 100 | 100 | 100 | 100 | 8.770001 | -12.26 | 57.52 |  |
| -22.97 | 100 | 100 | 100 | 57 | 9.1 | 13.93 | 100 |  |
| -7.6 | 100 | 100 | 100 | 60.55 | 55.54 | 0 | 100 |  |
| -4.45564 | 92.25745 | 67.50182 | 46.98709 | 46.908 | 41.88055 | 22.95164 | 48.44982 | Avr |
| 3.304677 | 2.837158 | 5.478477 | 5.102708 | 4.89844 | 4.988921 | 3.721245 | 4.388047 | SEM |

| Saline 2 | Saline 4 | Saline 6 | Saline 8 | Saline 10 | Saline 12 |  |
| --- | --- | --- | --- | --- | --- | --- |
| -3.73 | 2.72 | -7.5 | -27.3 | -5.31 | -4.28 |  |
| -3.5 | -1.05 | -3.91 | -5.14 | -14.77 | -0.98 |  |
| -1.08 | 2.75 | -4.25 | -8.79 | -18.6 | -12.22 |  |
| -10.68 | -0.4 | -2.65 | -5.08 | -6.28 | -3.42 |  |
| -12.85 | 4.18 | 0.61 | 10.26 | 14.44 | -3.26 |  |
| 8.83 | -1.69 | 3.6 | -8.99 | 5.1 | 2.28 |  |
| -10.91 | 3.44 | 4.71 | 18.57 | 1.98 | 3.62 |  |
| -21.18 | -12.45 | -6.22 | 6.54 | -10.5 | -10.22 |  |
| -14.36 | -17.79 | 8.74 | 2.64 | 0.6 | -4.78 |  |
| -14.96 | -24.85 | -12.73 | 8.92 | -25.78 | -3.96 |  |
| -11.23 | -11.79 | -20.12 | -14.12 | -5.74 | 3.62 |  |
| -6.74 | 3.57 | 1.53 | -5.16 | 3.38 | -14.22 |  |
| 0.33 | -14.08 | -2.54 | 2.43 | 6.4 | 2.4 |  |
| -7.85077 | -5.18769 | -3.13308 | -1.94 | -4.23692 | -3.49385 | Avr. |
| 2.196318 | 2.700347 | 2.11112 | 3.318555 | 3.098151 | 1.630233 | SEM |

**Experiment 2-D**

| Experimental Box | Morphine 6 | Test Box | Morphine 7 |  |
| --- | --- | --- | --- | --- |
| -10.54 | -0.84 | -3.27 | 63.97 |  |
| -4.32 | 11.35 | -3.74 | 65.14 |  |
| 3.33 | 35.13 | 0.82 | 9.95 |  |
| 6.6 | 39.12 | -21.9 | 32.02 |  |
| -13.3 | 87.55 | 2.46 | 54.29 |  |
| -12.85 | 61.91 | 1.5 | 41.78 |  |
| 13.03 | 25.08 | -9.85 | 72.9 |  |
| -10.11 | 38.46 | -1.19 | 90.61 |  |
| -6.86 | 19.53 | -8.27 | 54.08 |  |
| 2.3 | 97.06 | -9.43 | 60.06 |  |
| -9.93 | 13.75 | -7.05 | 85.87 |  |
| -18.34 | 24.68 | -16.03 | 33.09 |  |
| -0.32 | 15.63 | 6.5 | 51.79 |  |
| -6.79 | 25.51 | -8.13 | 28.74 |  |
| -8.86 | 15.47 | -4.06 | 31.8 |  |
| -8.15 | 8.31 | -9.85 | 39.96 |  |
| -6.99 | 68.84 | -1.19 | 100 |  |
| -15.32 | 100 | -8.27 | 70.84 |  |
| -0.92 | 83.98 | -3.87 | 100 |  |
| -8.35 | 36.84 | 1.55 | 30.33 |  |
| 0.83 | 17.47 | -11.33 | 89.23 |  |
| -9.05 | 9.9 | -9.43 | 73.86 |  |
| -1.84 | 3.56 | -7.05 | 19.59 |  |
| -2.17 | 2.82 | -16.03 | 37.32 |  |
| 2.77 | 15.63 | 33.19 | 51.79 |  |
| -5.69 | 25.51 | 6.52 | 28.74 |  |
| -8.86 | 15.47 | -12.92 | 31.8 |  |
| -8.15 | 8.31 | -5.42 | 39.96 |  |
| -6.99 | 68.84 | -17.77 | 100 |  |
| -15.32 | 100 | -4.3 | 70.84 |  |
| -17.65 | 100 | 3.13 | 95.66 |  |
| -15.32 | 72.4 | 2.66 | 40.8 |  |
| -18.49 | 60 | 5.44 | 100 |  |
| 6.96 | 72.16 | -2.54 | 71.25 |  |
| 13.1 | 100 | -1.24 | 100 |  |
| 8.61 | 27.77 | -0.41 | 100 |  |
| -40.97 | 35.87 | -22.13 | 22.64 |  |
| 17.41 | 17.48 | -48.35 | 26.58 |  |
| -23.9 | 35.55 | -6.32 | 39.19 |  |
| -18.63 | 43.58 | 2.3 | 51.6 |  |
| -14.5 | 21.31 | -18.02 | 59.4 |  |
| -27.92 | 53.62 | -17.09 | 68.53 |  |
|  | 76.03 | 6.97 | 32.83 |  |
|  | 25.4 | -5.57 | 76.14 |  |
|  | 79.28 | -13.46 | 22.39 |  |
|  | 23.88 | 16.35 | 88.96 |  |
|  | 9.81 | 0.76 | 69.8 |  |
|  | 26.78 | -7.02 | 65.71 |  |
|  |  | -13.27 |  |  |
|  |  | -6.98 |  |  |
|  |  | 3.54 |  |  |
|  |  | -2.93 |  |  |
|  |  | 0.22 |  |  |
|  |  | -10.18 |  |  |
| -7.20143 | 40.33596 | -5.2213 | 57.27298 | Avr. |
| 1.75173 | 4.549519 | 1.516605 | 3.780872 | SEM |

|  |  |  |  |
| --- | --- | --- | --- |
|  |  |  |  |

**Experiment 2-E**

| Experimental Box | Morphin 6 | Test Box | Morphine 7 |  |
| --- | --- | --- | --- | --- |
| -2.78 | -7.74 | -20.3 | 53.04 |  |
| 6.86 | 100 | 7.65 | 46.99 |  |
| -7.43 | 62.73 | -17.63 | 11.37 |  |
| 2.46 | 28.54 | -29.53 | 49.24 |  |
| -10.25 | 25.47 | -12.11 | 63.3 |  |
| -11.23 | 30.6 | 3.11 | 71.49 |  |
| 7.69 | 42.22 | -12.34 | 42.33 |  |
| 0.95 | 87.17 | -5.3 | 100 |  |
| -4.22 | 15.89 | -17.85 | 99.58 |  |
| -3.88 | 75.64 | -10.84 | 38.3 |  |
| -6.06 | 38.95 | -0.73 | 99.13 |  |
| -14.7 | 34.37 | -12.79 | 5.82 |  |
| -3.33 | 29.88 | 3.7 | 58.84 |  |
| -7.01 | 13.5 | -4.93 | 40.57 |  |
| -4.84 | 19.92 | -16.38 | 53.59 |  |
| -8.89 | -1.4 | -12.34 | 50.9 |  |
| -6.72 | 100 | -5.3 | 95.68 |  |
| -8.38 | 20.81 | -17.85 | 34.14 |  |
| -3.62 | -6.47 | -0.13 | 77.31 |  |
| 7.58 | 32.07 | 0.39 | 21.03 |  |
| -0.06 | 17.88 | -5.34 | 84.42 |  |
| 1.22 | 10.34 | -10.84 | 100 |  |
| -1.65 | 1.94 | -0.73 | 11.34 |  |
| 4.4 | 4.2 | -12.79 | 67.22 |  |
| -0.15 | 29.88 | 47.35 | 58.84 |  |
| -5.91 | 13.5 | -18.23 | 40.57 |  |
| -4.84 | 28.57 | -16.28 | 53.59 |  |
| -8.89 | -1.77 | -13.27 | 51.1 |  |
| -6.72 | 100 | -20.52 | 95.68 |  |
| -8.38 | 24.31 | -4.02 | 34.14 |  |
| -14.67 | 100 | -0.06 | 91.15 |  |
| -11.95 | 100 | 0.06 | 77.04 |  |
| -15.13 | 100 | 1.65 | 100 |  |
| -16.2 | 52.7 | 3.23 | 90.5 |  |
| -7.31 | 68.97 | -2.84 | 82.11 |  |
| -1.27 | 38.95 | 1.55 | 100 |  |
| -4.1 | 32.19 | -34.09 | 41.54 |  |
| -20.22 | 11.48 | -57.86 | 26.13 |  |
| -21.4 | 100 | -9.55 | 48.96 |  |
| -1.81 | 14.01 | -4.42 | 56.86 |  |
| -19.28 | 27.08 | -17.64 | 69.99 |  |
| -38.51 | 23.81 | -15.8 | 71.29 |  |
| -17.28 | 67.09 | 1.88 | 95.29 |  |
| -6.6 | 40.32 | 4.65 | 100 |  |
| -48.54 | 13.59 | -7.76 | 38.9 |  |
| -9.89 | 11.69 | 2.04 | 29.9 |  |
| -30.64 | 4.04 | -2.28 | 56.36 |  |
| -16.83 | 2.71 | -10.6 | 39.45 |  |
| -16.59 | -7.8 | -6.56 | 22.54 |  |
| -13.16 | 5.42 | -9 | 37.77 |  |
|  | 94.24 | -11.26 | 45.43 |  |
|  | 36.23 | -14.46 | 95.15 |  |
|  | 43.68 | 2.33 | 42.2 |  |
|  | 6.3 | -11.97 | 100 |  |
|  | -0.24 | -18.58 | 72.61 |  |
|  | 32.02 | -7.73 | 100 |  |
|  |  | -3.6 |  |  |
|  |  | -22.02 |  |  |
|  |  | -4.41 |  |  |
|  |  | 2.56 |  |  |
| -8.8032 | 35.52643 | -8.14517 | 61.44143 | Avr. |
| 1.505871 | 4.495143 | 1.691428 | 3.719118 | SEM |

**Experiment 2-F**

| Experimental Box | Morphine 6 | Test Box | Morphine 7 |  |
| --- | --- | --- | --- | --- |
| 3.53 | -4.85 | -4.78 | 46.26 |  |
| -4.09 | -2.64 | -0.3 | 19.97 |  |
| -9.05 | 8.38 | -13.97 | 83.49 |  |
| 8.16 | 40.75 | 5.28 | 44.99 |  |
| -14.56 | 17.35 | -9.57 | 42.61 |  |
| -8.31 | 38.75 | 4.49 | 36.98 |  |
| 12.03 | -10.32 | -9.05 | 56.38 |  |
| 5.22 | 11.22 | 11.26 | 65.29 |  |
| -9.15 | 20.58 | -21.12 | 12.3 |  |
| 3.68 | 23.9 | -12.47 | 59.4 |  |
| -9.51 | 30.83 | -2.03 | 49.46 |  |
| -12.4 | 9.95 | -16.48 | 22.15 |  |
| -9.21 | 14.2 | 11.63 | 59.76 |  |
| -6.19 | 19.1 | -16.27 | 56.43 |  |
| -10.65 | 12.85 | -11.59 | 8.41 |  |
| -8.96 | 7.87 | -9.05 | 27.12 |  |
| -5.91 | 88.26 | 11.26 | 100 |  |
| -0.87 | 13.79 | -21.12 | 21.31 |  |
| 1.84 | 22.65 | -1.67 | 100 |  |
| 4.63 | -3.58 | 8.91 | 2.39 |  |
| -1.22 | 12.55 | -7.34 | 94.94 |  |
| -7.29 | 51.88 | -12.47 | 100 |  |
| 1.4 | 9.42 | -2.03 | 6.42 |  |
| 2.1 | -0.92 | -16.48 | 29.05 |  |
| -5.85 | 14.2 | 14.01 | 59.76 |  |
| -5.1 | 19.1 | -6.16 | 56.43 |  |
| -10.65 | 12.78 | -9.56 | 8.41 |  |
| -8.96 | 7.87 | -14.48 | 27.12 |  |
| -5.91 | 88.26 | -14.41 | 100 |  |
| -0.87 | 13.79 | -0.76 | 21.31 |  |
| -4.81 | 70.7 | 3.07 | 100 |  |
| -8.75 | 65.55 | 2.09 | 42.99 |  |
| -15.04 | 80 | 0 | 0 |  |
| 0 | 76.69 | 2.85 | 67.92 |  |
| -8.1 | 100 | 4.34 | 100 |  |
| -2.25 | 47 | -0.79 | 100 |  |
| 105.77 | 29.53 | -27.38 | 12.24 |  |
| -3.91 | 15.28 | -54.55 | 44.25 |  |
| 107.63 | 29.36 | -9.41 | 28.02 |  |
| -19.82 | 37.08 | -4.1 | 28.41 |  |
| -25.72 | 5.79 | -18.39 | 36.43 |  |
| 13.43 | 14.15 | -19.61 | 54.49 |  |
| -0.43 | 4.18 | 0.4 | 36.12 |  |
| -19.85 | 7.81 | -0.56 | 100 |  |
| -29.41 | 13.46 | -3.57 | 17.61 |  |
| -16.61 | 12.39 | 1.68 | 11.36 |  |
| -17.52 | 0.96 | 0.83 | -6.66 |  |
| -32.63 | 2.39 | -3.73 | -8.49 |  |
| -19.18 | -10.47 | -3.5 | 25.99 |  |
| -3.09 | 3.32 | -1.08 | 20.74 |  |
| -50.05 | 100 | -10.68 | 71.34 |  |
| -8.14 | 35.47 | -12.85 | 60.54 |  |
| -20.4 | 12.06 | 8.83 | 45.78 |  |
| -23.49 | -12.26 | -10.91 | 57.52 |  |
| -22.97 | 13.93 | -21.18 | 100 |  |
| -7.6 | 0 | -14.36 | 100 |  |
|  |  | -14.96 |  |  |
|  |  | -11.23 |  |  |
|  |  | -6.74 |  |  |
|  |  | 0.33 |  |  |
|  |  |  |  |  |
|  |  |  |  |  |
| -4.37607 | 23.97036 | -6.52467 | 47.58464 | Avr |
| 3.275481 | 3.827865 | 1.565483 | 4.43475 | SEM |
