# Supplementary material for: Unconditioned and learned morphine tolerance influence hippocampal-dependent short-term memory and the subjacent expression of GABA-A receptor alpha subunits
Source: PLoS One. 2021 Sep 9;16(9):e0253902. doi: 10.1371/journal.pone.0253902 (PMC8428970; doi:10.1371/journal.pone.0253902)
Supplement: S3 File — Experiment 3. (DOCX) [file pone.0253902.s007.docx]

**Appendix 3.** Fig 3, the effects of the expression of morphine tolerance and VPA pre-treatment on STM, assessed using the Y-maze apparatus with a 1 h ITI.

**Experiment 3-A**

| S | VPA | NAMT | NAMTV | AMT | AMTV |  |
| --- | --- | --- | --- | --- | --- | --- |
| 0 | 0.88 | 1.7 | 1.6 | 9.96 | 2.12 |  |
| 0 | 1.61 | 7.9 | 0.9 | 4.05 | 3.21 |  |
| 6.3 | 0 | 2.3 | 1.2 | 0 | 1.82 |  |
| 0 | 0.8 | 0.9 | 4 | 3.6 | 0 |  |
| 0 | 3.31 | 0 | 2.6 | 15.02 | 1.62 |  |
| 1.44 | 0 | 1.24 | 0 | 2.36 |  |  |
| 0 | 2.65 | 0.75 | 1.7 | 3.5 |  |  |
| 0.75 | 4 | 0.91 | 1.51 | 17.86 |  |  |
| 1.65 | 0.92 | 1.51 | 0.85 | 1.26 |  |  |
| 0.83 | 1.58 | 1.38 | 0 |  |  |  |
| 1.097 | 1.575 | 1.859 | 1.436 | 6.4011111 | 1.754 | Avr. |
| 0.61195 | 0.428455 | 0.698839 | 0.377737 | 2.120611 | 0.517335 | SEM |

**Experiment 3-B**

|  | Novel arm | | | | | | | | | | Other arm | | | | | | | | | | Initial arm | | | | | | | | | |
| --- | --- | --- | --- | --- | --- | --- | --- | --- | --- | --- | --- | --- | --- | --- | --- | --- | --- | --- | --- | --- | --- | --- | --- | --- | --- | --- | --- | --- | --- | --- |
| TC | 57.14 | 40 | 50 | 50 | 36.36 | 50 | 57.14 | 42.86 | 50 | 40 | 28.57 | 30 | 25 | 25 | 36.36 | 25 | 28.57 | 28.57 | 20 | 40 | 14.29 | 30 | 25 | 25 | 27.27 | 25 | 14.29 | 28.57 | 30 | 20 |
| V | 50 | 66.67 | 50 | 42.86 | 50 | 50 | 28.57 | 33.33 | 37.5 | 33.33 | 16.67 | 33.33 | 20 | 28.57 | 16.67 | 30 | 28.57 | 44.44 | 37.5 | 33.33 | 33.33 | 0 | 30 | 28.57 | 33.33 | 20 | 42.86 | 22.22 | 25 | 33.33 |
| NAMT | 42.86 | 36.36 | 20 | 33.33 | 44.44 | 20 | 40 | 42.86 | 33.33 | 40 | 28.57 | 27.27 | 40 | 33.33 | 33.3 | 40 | 20 | 28.57 | 33.33 | 40 | 28.57 | 27.27 | 40 | 33.33 | 22.22 | 40 | 40 | 28.57 | 33.33 | 20 |
| NAMTV | 33.33 | 23.08 | 31.25 | 33.33 | 30.77 | 50 | 40 | 42.86 | 40 | 44.44 | 33.33 | 38.46 | 37.5 | 41.67 | 30.77 | 33.33 | 40 | 28.57 | 20 | 33.3 | 33.33 | 38.46 | 31.25 | 25 | 38.46 | 16.67 | 20 | 28.57 | 40 | 22.22 |
| AMT | 40 | 30.77 | 46.15 | 30 | 31.57 | 25 | 42.1 | 30.76 | 30.76 | 34.61 | 30 | 30.77 | 23.08 | 30 | 26.31 | 25 | 26.31 | 30.76 | 30.76 | 30.76 | 30 | 38.46 | 30.77 | 40 | 50 | 31.57 | 38.46 | 30.76 | 30.76 | 21.77778 |
| AMTV | 55.45 | 31.25 | 59.85 | 33.33 | 48.88 | 0 | 0 | 0 | 0 | 0 | 27.27 | 31.25 | 28.57 | 33.33 | 33.33 | 0 | 0 | 0 | 0 | 0 | 27.27 | 27.5 | 28.57 | 31.33 | 0 | 0 | 0 | 0 | 0 | 0 |

**Experiment 3-C**

|  | Novel arm | | | | | | | | | | Other arm | | | | | | | | | | Initial arm | | | | | | | | | |
| --- | --- | --- | --- | --- | --- | --- | --- | --- | --- | --- | --- | --- | --- | --- | --- | --- | --- | --- | --- | --- | --- | --- | --- | --- | --- | --- | --- | --- | --- | --- |
| S | 54.77 | 43.54 | 50.7 | 27.37 | 39 | 31.42 | 39.66 | 58.05 | 27.7 | 36.47 | 23.49 | 27.7 | 34.3 | 32.25 | 54.14 | 25.7 | 31.82 | 26.4 | 59.27 | 33.97 | 41.74 | 18.76 | 25 | 20.38 | 26.86 | 42.88 | 28.52 | 15.55 | 13.03 | 29.56 |
| V | 46.34 | 37 | 29.19 | 25.38 | 37.16 | 14.85 | 51.99 | 46.48 | 33.34 | 67.76 | 35.47 | 33.64 | 33.43 | 34.08 | 28.8 | 52.9 | 16.29 | 22.22 | 24.53 | 21.36 | 18.19 | 29.37 | 37.39 | 40.54 | 34.04 | 32.25 | 31.72 | 31.3 | 42.13 | 10.88 |
| NAMT | 10.48 | 50.03 | 48.98 | 15.06 | 36.59 | 33.29 | 19.89 | 29.97 | 16.38 | 24.82 | 68.55 | 36.72 | 31.13 | 50.72 | 33.96 | 25.12 | 44.96 | 27.73 | 49.63 | 31.73 | 20.97 | 13.24 | 19.89 | 34.22 | 29.44 | 41.59 | 35.15 | 42.3 | 33.99 | 43.45 |
| NAMTV | 55.56 | 16.86 | 28.45 | 21.54 | 34.84 | 30.77 | 38.46 | 45.45 | 31.58 | 33.33 | 22.22 | 37.99 | 28.77 | 40 | 32.82 | 38.46 | 38.46 | 36.36 | 31.58 | 38.89 | 22.22 | 45.14 | 42.78 | 38.46 | 32.34 | 30.77 | 23.08 | 18.18 | 36.84 | 27.78 |
| AMT | 0 | 0 | 0 | 35.03 | 32.23 | 51.75 | 20.61 | 24.3 | 0 | 41.57 | 44.64 | 28.3 | 23.15 | 30.95 | 25 | 0 | 24.71 | 49.03 | 11.23 | 39.5 | 20.34 | 39.47 | 25.1 | 48.44 | 44.82 | 100 | 33.7 | 28.84 | 41.57 | 16.04 |
| AMTV | 59.39 | 33.55 | 50.13 | 25.35 | 49.73 | 0 | 0 | 0 | 0 | 0 | 19.84 | 31.88 | 28.14 | 20.75 | 22.54 | 0 | 0 | 0 | 0 | 0 | 20.76 | 34.55 | 21.7 | 53.88 | 27.72 | 0 | 0 | 0 | 0 | 0 |
