# Supplementary material for: Unconditioned and learned morphine tolerance influence hippocampal-dependent short-term memory and the subjacent expression of GABA-A receptor alpha subunits
Source: PLoS One. 2021 Sep 9;16(9):e0253902. doi: 10.1371/journal.pone.0253902 (PMC8428970; doi:10.1371/journal.pone.0253902)
Supplement: S4 File — Experiment 4. (DOCX) [file pone.0253902.s008.docx]

| S | VPA | NAMT | NAMTV | AMT | AMTV |  |
| --- | --- | --- | --- | --- | --- | --- |
| 57.14 | 50 | 42.86 | 33.33 | 44.44 | 45.45455 |  |
| 40 | 66.67 | 36.36 | 23.08 | 30.77 | 31.25 |  |
| 50 | 50 | 20 | 31.25 | 46.15 | 42.85714 |  |
| 50 | 42.86 | 33.33 | 33.33 | 30 | 33.33333 |  |
| 36.36 | 50 | 44.44 | 30.77 | 33.33 | 38.88889 |  |
| 50 | 50 | 20 | 50 | 30.77 | 27.27273 |  |
| 57.14 | 28.57 | 40 | 40 | 23.08 | 31.25 |  |
| 42.86 | 33.33 | 42.86 | 42.86 | 30 | 28.57143 |  |
| 50 | 37.5 | 33.33 | 40 | 22.22 | 33.33333 |  |
| 40 | 33.33 | 40 | 44.44 | 38.46 | 33.33333 |  |
| 28.57 | 16.67 | 28.57 | 33.33 | 30.77 | 27.27273 |  |
| 30 | 33.33 | 27.27 | 38.46 | 40 | 37.5 |  |
| 25 | 20 | 40 | 37.5 |  | 28.57143 |  |
| 25 | 28.57 | 33.33 | 41.67 |  | 33.33333 |  |
| 36.36 | 16.67 | 33.3 | 30.77 |  | 27.77778 |  |
| 25 | 30 | 40 | 33.33 |  |  |  |
| 28.57 | 28.57 | 20 | 40 |  |  |  |
| 28.57 | 44.44 | 28.57 | 28.57 |  |  |  |
| 20 | 37.5 | 33.33 | 20 |  |  |  |
| 40 | 33.33 | 40 | 33.3 |  |  |  |
| 14.29 | 50 | 28.57 | 33.33 |  |  |  |
| 30 | 66.67 | 27.27 | 38.46 |  |  |  |
| 25 | 50 | 40 | 31.25 |  |  |  |
| 25 | 42.86 | 33.33 | 25 |  |  |  |
| 27.27 | 50 | 22.22 | 38.46 |  |  |  |
| 25 | 50 | 40 | 16.67 |  |  |  |
| 14.29 | 28.57 | 40 | 20 |  |  |  |
| 28.57 | 33.33 | 28.57 | 28.57 |  |  |  |
| 30 | 37.5 | 33.33 | 40 |  |  |  |
| 20 | 33.33 | 20 | 22.22 |  |  |  |
| 33.333 | 39.12 | 33.028 | 33.33167 | 33.3325 | 33.33333 | Avr. |
| 2.182852 | 2.307474 | 1.383688 | 1.451866 | 2.18279 | 1.366173 | SEM |

**Appendix 4.** Fig 4, The effect of the expression of morphine tolerance and VPA pre-treatment on SWM.

**Experiment 4-A**

**Experiment 4-B**

| Saline | VPA | NAMT | NAMTV | AMT | AMTV |  |
| --- | --- | --- | --- | --- | --- | --- |
| 71.43 | 56.25 | 64.29 | 80 | 43.72 | 80 |  |
| 72.41 | 73.91 | 59.09 | 55.56 | 43.3 | 64.7 |  |
| 58.33 | 66.67 | 80 | 62.07 | 24.16 | 48.14 |  |
| 78.57 | 57.89 | 66.66 | 64.29 | 39.23 | 64.28 |  |
| 63.64 | 64.29 | 76.92 | 71.43 |  | 45.83 |  |
| 65 | 100 | 88 | 58.33 |  |  |  |
| 66.67 | 59.09 | 43.75 | 71.43 |  |  |  |
| 83.33 | 45.45 | 70 | 72.22 |  |  |  |
| 64.29 | 70 | 60 | 61.9 |  |  |  |
| 80 | 69.56 | 75 | 75 |  |  |  |
| 70.367 | 66.311 | 68.371 | 67.223 | 37.6025 | 60.59 | Avr. |
| 2.588374 | 4.571084 | 3.975201 | 2.50045 | 4.59379 | 6.245464 | SEM |
