# Supplementary material for: Unconditioned and learned morphine tolerance influence hippocampal-dependent short-term memory and the subjacent expression of GABA-A receptor alpha subunits
Source: PLoS One. 2021 Sep 9;16(9):e0253902. doi: 10.1371/journal.pone.0253902 (PMC8428970; doi:10.1371/journal.pone.0253902)
Supplement: S5 File — Experiment 5. (DOCX) [file pone.0253902.s009.docx]

**Appendix 5.** Fig 5, Quantification data for qRT-PCR analysis of changes in the expression of *Gabrα*1, α2 and α5 subunits in the hippocampus of rats.

**Experiment 5-A**

| S | VPA | NAMT | NAMTV | AMT | AMTV |  |
| --- | --- | --- | --- | --- | --- | --- |
| 1 | 0.13 | 1.79 | 0.34 | 1.77 | 1.32 |  |
| 1 | 0.78 | 1.96 | 1.15 | 2.26 | 1.26 |  |
| 1 | 0.26 | 2.18 | 0.64 | 2.33 | 2.88 |  |
| 1 | 0.01 | 1.72 | 0.21 | 2.16 | 1.18 |  |
| 1 | 0.03 | 2.03 | 0.74 | 1.87 | 2.01 |  |
| 1 | 0.15 | 1.5 | 0.13 | 2.65 | 2.36 |  |
| 1 | 0.2266667 | 1.8633333 | 0.535 | 2.1733333 | 1.835 | Avr. |
| 0 | 0.1166381 | 0.0992192 | 0.1569448 | 0.1309113 | 0.2842036 | SEM |

**Experiment 5-B**

| S | VPA | NAMT | NAMTV | AMT | AMTV |  |
| --- | --- | --- | --- | --- | --- | --- |
| 1 | 0.1 | 1.239825 | 0.26 | 1.11 | 2.23 |  |
| 1 | 0.44 | 1.470883 | 0.14 | 1.38 | 1.82 |  |
| 1 | 0.16 | 1.220413 | 0.1 | 3.12 | 2.51 |  |
| 1 | 0.05 | 1.25861 | 0.11 | 2.75 | 2.21 |  |
| 1 | 0.04 | 1.195366 | 0.87 | 2.01 | 1.61 |  |
| 1 | 0.04 | 2.281054 | 0.15 | 3.27 | 2.56 |  |
| 1 | 0.15 | 0.940911 |  | 3.25 |  |  |
| 1 | 0.45 | 0.962572 |  | 4.53 |  |  |
| 1 |  | 1.268003 |  | 0.85 |  |  |
| 1 |  | 1.307451 |  | 1.11 |  |  |
| 1 |  | 1.555416 |  |  |  |  |
| 1 | 0.17875 | 1.336409 | 0.271667 | 2.338 | 2.156667 | Avr. |
| 0 | 0.060399 | 0.10908 | 0.121913 | 0.3879 | 0.153616 | SEM |

**Experiment 5-C**

| S | VPA | NAMT | NAMTV | AMT | AMTV |  |
| --- | --- | --- | --- | --- | --- | --- |
| 1 | 0.46 | 1.63 | 0.51 | 1.31 | 0.16 |  |
| 1 | 0.08 | 2.54 | 0.49 | 3.37 | 1.38 |  |
| 1 | 0.15 | 1.72 | 0.9 | 3.16 | 1.55 |  |
| 1 | 0.37 | 1.23 | 0.27 | 2.86 | 2.62 |  |
| 1 | 0.11 | 2 | 0.42 | 2.03 | 1.08 |  |
| 1 | 0.52 | 1.17 | 0.13 | 2.75 | 1.39 |  |
| 1 | 0.281667 | 1.715 | 0.453333 | 2.58 | 1.363333 | Avr. |
| 0 | 0.078291 | 0.208275 | 0.106979 | 0.315341 | 0.323694 | SEM |
