# Supplementary material for: Unconditioned and learned morphine tolerance influence hippocampal-dependent short-term memory and the subjacent expression of GABA-A receptor alpha subunits
Source: PLoS One. 2021 Sep 9;16(9):e0253902. doi: 10.1371/journal.pone.0253902 (PMC8428970; doi:10.1371/journal.pone.0253902)
Supplement: S6 File — Experiment 6. (DOCX) [file pone.0253902.s010.docx]

**Appendix 6.** Fig 6, The effects of morphine tolerance and VPA pretreatment on hippocampal *Gabrα* protein density was separated on SDS–PAGE, western blotted, probed with specific primary antibody, and reported with anti β-actin antibody

| S | VPA | NAMT | NAMTV | AMT | AMTV |  |
| --- | --- | --- | --- | --- | --- | --- |
| 0.946236 | 0.214596 | 1.225067 | 0.472335 | 2.35964 | 1.238242 |  |
| 0.925346 | 0.279544 | 1.203921 | 0.573526 | 2.617017 | 1.577556 |  |
| 0.605038 | 0.405013 | 1.691437 | 0.390314 | 1.392923 | 0.488423 |  |
| 0.716202 | 0.358629 | 1.320421 | 0.490304 | 1.489496 | 1.326451 |  |
| 1.137538 | 0.255793 | 1.408033 | 0.67677 | 1.631081 | 1.260161 |  |
| 1.117052 | 0.302722 | 1.502879 | 0.488992 | 2.15452 | 1.618591 |  |
|  |  |  |  |  |  |  |
| 0.907902 | 0.302716 | 1.39196 | 0.515374 | 1.94078 | 1.251571 | Avr. |
| 0.086934 | 0.02834 | 0.075405 | 0.040103 | 0.206409 | 0.166255 | SEM |
