# Supplementary material for: Unconditioned and learned morphine tolerance influence hippocampal-dependent short-term memory and the subjacent expression of GABA-A receptor alpha subunits
Source: PLoS One. 2021 Sep 9;16(9):e0253902. doi: 10.1371/journal.pone.0253902 (PMC8428970; doi:10.1371/journal.pone.0253902)
Supplement: Attachment — Submitted filename: responses to reviewers.docx [file pone.0253902.s012.docx]

MPORTANT: PLEASE DO NOT REPLY TO THIS EMAIL
If you are unable to complete any points that are requested in this email, please explain why in the "Enter Comments" tab of the online submission form prior to re-submitting your manuscript.  This will enable us to promptly assess your response and progress your manuscript to an Academic Editor at the earliest opportunity.
=============================================

PONE-D-20-29685R1
Unconditioned and learned morphine tolerance influence hippocampal-dependent short-term memory and the subjacent expression of GABA-A receptor alpha subunits
Prof. Yaghoub Fathollahi

Dear Prof. Fathollahi,

Thank you for submitting your manuscript entitled "Unconditioned and learned morphine tolerance influence hippocampal-dependent short-term memory and the subjacent expression of GABA-A receptor alpha subunits" to PLOS ONE.  Your manuscript files have been checked in-house but before we can proceed we need you to address the following issues:

1. In the Method section of your manuscript, please address the following concerns regarding the ethical handling of animals in your study:

a) Please explicitly state that the IACUC/animal ethics committee specifically approved your study protocol, and provide their name.

It was applied at the manuscript: P5p2l1-5

The project was found to be accordance to ethical principles and the national norms and standards for conducting Medical Research at Tarbiat modares university and Ministry of Health and Medical Education of Iran (ID: IR.TMU.REC.1396.563)

b) Please provide the ID number for your approved study protocol.

It was applied at the manuscript: P5p2l5
(ID: IR.TMU.REC.1396.563)

c) Please specify the method of sacrifice used, as well as who performed the sacrifice (e.g. authors, licensed veterinarians, etc.).

It was applied at the manuscript: P6p1l3-5

For euthanasia the compressed carbon dioxide gas was used due to its rapid onset of action, safety and availability. Then the rats were decapitated with the rodent guillotine. Animals were scarified by the authors (Gh.Ghamkharinejad- S.H.Marashi).

2. We note your Data Availability Statement currently reads as follows:
"in our research lab."

We also note a number of Supporting Information files uploaded to your submission, which appear to contain data used for your study.

Before we proceed, we’ll require some additional clarification to ensure your submission adheres to the PLOS ONE Data Availability policy (<https://journals.plos.org/plosone/s/data-availability>).

1.) Please confirm whether the data currently located in your manuscript and/or Supporting Information files constitute the minimal data set, defined as the data set used to reach the conclusions drawn in the manuscript with related metadata and methods, and any additional data required to replicate the reported study findings in their entirety. This may include:

a.) The values behind the means, standard deviations and other measures reported;

b.) The values used to build graphs;

c.) The points extracted from images for analysis (<https://journals.plos.org/plosone/s/data-availability#loc-minimal-data-set-definition>).

We prepared supporting data files (Raw-data for graph 1-6, Supplementary graph 1 and 2, Raw-blot image) consist of means, standard deviations and the values used to build graphs.

Raw-data for graph 1

<https://gin.g-node.org/Ghazaleh-Ghamkharinejad/PONE-D-20-29685R1/commit/5ec65191342c636b5394558b6fee3cfcce2fa8ae>

Raw-data for graph 2

<https://gin.g-node.org/Ghazaleh-Ghamkharinejad/PONE-D-20-29685R1/commit/5ec65191342c636b5394558b6fee3cfcce2fa8ae>

Raw-data for graph 3

<https://gin.g-node.org/Ghazaleh-Ghamkharinejad/PONE-D-20-29685R1/commit/5ec65191342c636b5394558b6fee3cfcce2fa8ae>

Raw-data for graph 4

<https://gin.g-node.org/Ghazaleh-Ghamkharinejad/PONE-D-20-29685R1/commit/5ec65191342c636b5394558b6fee3cfcce2fa8ae>

Raw-data for graph 5

<https://gin.g-node.org/Ghazaleh-Ghamkharinejad/PONE-D-20-29685R1/commit/5ec65191342c636b5394558b6fee3cfcce2fa8ae>

Raw-data for graph 6

<https://gin.g-node.org/Ghazaleh-Ghamkharinejad/PONE-D-20-29685R1/commit/5ec65191342c636b5394558b6fee3cfcce2fa8ae>

Supplementary graph 1

<https://gin.g-node.org/Ghazaleh-Ghamkharinejad/PONE-D-20-29685R1/commit/aa6d40475a973df08a7f315eaa5c5c94d94c4d71>

Supplementary graph 2

<https://gin.g-node.org/Ghazaleh-Ghamkharinejad/PONE-D-20-29685R1/commit/aa6d40475a973df08a7f315eaa5c5c94d94c4d71>

Raw-blot image

<https://gin.g-node.org/Ghazaleh-Ghamkharinejad/PONE-D-20-29685R1/commit/5ec65191342c636b5394558b6fee3cfcce2fa8ae>

2.) If the minimal data set is located in your manuscript and/or Supporting Information files, please confirm whether we may update your Data Availability Statement as follows:

“All relevant data are within the manuscript and its Supporting Information files.”

The Authors confirm all relevant data are within the manuscript and its Supporting Information files.”

3.) If additional data are required to fulfill the minimal data set, please either upload them as Supporting Information or to a stable public repository. Please also provide the relevant URLs, DOIs, or accession numbers for any data uploaded to a repository. For a list of acceptable repositories, please see <https://journals.plos.org/plosone/s/recommended-repositories>.

The Authors confirm all relevant data are available


Your manuscript has been returned to your account. Please log on to PLOS Editorial Manager at <https://www.editorialmanager.com/pone/> to access your manuscript.
