# Supplementary material for: Unconditioned and learned morphine tolerance influence hippocampal-dependent short-term memory and the subjacent expression of GABA-A receptor alpha subunits
Source: PLoS One. 2021 Sep 9;16(9):e0253902. doi: 10.1371/journal.pone.0253902 (PMC8428970; doi:10.1371/journal.pone.0253902)
Supplement: Attachment — Submitted filename: responses to comments.docx [file pone.0253902.s013.docx]

Answer to the reviewer comments:

General appointments


This is a re-review of the study of Fathollahi et al., now entitled “Unconditioned and learned morphine tolerance influence hippocampal-dependent short-term memory and the subjacent expression of GABA-A receptors alpha subunits”. Based on the previous reviewer’s criticisms the authors performed profound (and huge) changes to the article. These changes help to turn the manuscript more understandable. The study brings new and important information on the behavioral and neural events underlying morphine tolerance. However, after this second round of review, I think that the manuscript still needs to be improved to be considered for publication in PONE. In general, the manuscript lacks organization. My points to be considered by the authors are provided below:

Protocol DOI: http://dx.doi.org/10.17504/protocols.io.bveyn3fw

1. The title is now precise.

Thank you so much for consideration

2. My first criticism concerns the excessive number of abbreviations used in the manuscript. In my point of view, it is very annoying having to come back every time in the reading to remember each of the abbreviations used. This does not testify against the quality of the study but severely limited the enthusiasm of the reader. I think the authors must use only the most important acronyms, including NANT, AMT, STM, and SWM. I recommend that the authors use GABAr-α1 (or α2, and α5) instead of Gabr or Gabrs, since the word GABA itself is an acronym.

It was applied at the whole manuscript

4. Another concern about the study is the absolute absence of a topic informing the total number of animals used (including losses, if any), and the number of animals by group subdivisions. In other words, how many groups were used and how many rats belonging to each group. This is important to the reader considering that any inference on the statistical analysis requires such information and this could be done through the presentation of a schematic picture of the procedures used in the study. In fact, considering the number of different dependent variables collected, this is necessary for each one of the experimental procedures used, including the STM and SWM.

In some figures related to STM and SWM parameters, the number of animals inserted into the bar charts. A total number of 190 rats (including losses) were used in this study (morphine tolerance development, SWM, STM and molecular tests). It should be mentioned that different behavioral and molecular assessments were carried out on same groups of rats by the same experimenter. For evaluating the morphine tolerance development and analgesic response to VPA, 186 rats were categorized into six groups according to the following order: groups 1 and 2 (saline and VPA exposure, *n* = 30 for each group); groups 3 and 4 (non- associative morphine tolerance and non-associative morphine tolerance +VPA, n=36 and 30, respectively); groups 5 and 6 (associative morphine tolerance and associative morphine tolerance +VPA, n=48 and 12, respectively) (Supplementary Figures 1, 2 and Figure 1 and 2)). 60 rats were used for short term learning analyses (N= 10 rats per group) and 147 rats were used for working memory assessment (N= 30, 30, 30, 30, 12, 15 for groups 1-6, respectively (Figures 3 and 4). Finally, 36 rats were used for mRNA expression and protein level assessments (n=6 in each group).

P6-7p4

5. On page 7, line 16. How long habituation to the new context lasts before saline injections?

It was done at P8p1l6

6. Page 9, line 14. After the word “method”, insert the corresponding reference.

It was done at the manuscript at P1p1l8

7. Page 18, last line, after Griesel et al., the year of publication is missing.

It was applied at the manuscript at P20p1l2

8. Page 19, line 23 (We observed no significant...). Looking at figure 3, It seems to me that the author’s assumption does not correlate with the information posted in the figure. If I understand the point, considering that the overall locomotion is correlated with the percentage of arm entries, both NAMT and AMT groups have reduced (and not increased) novel arm entries. Therefore, morphine decreases novel arm exploration of NAMT and AMT groups, which is reversed by valproate. Perhaps may this be due to my inability to understand the author's inference but the manuscript can largely benefit from a more organized way of discussing the data. I suggest dividing both the results and the discussion section into behavioral (morphine tolerance, short-term memory, spatial-working memory) and molecular topics.

Thank you for your consideration and accuracy. You are absolutely right and as you carefully indicated the locomotion didn’t change significantly in different groups (Fig4, and result part p17p1, line 1). We made a mistake in writing of mentioned sentences in the discussion part. In the revised version, we corrected them (Discussion part p21p2l, line 1). Also, we reviewed carefully the entire results and discussion to organize these parts better. We tried to divide the result and discussion to behavioral and molecular sub-sessions and it was applied in manuscript, since the PLOS journal does not allow to add sub-session into the discussion, we added few sentences in the first paragraph of each behavioral and molecular part of discussion.

9. Page 20, line 8 (In accordance with...). I was not able to find such data in the present report.

It was edited at P21p3l6

10. Page 21, line 14. Pervious (previous?) documents were reviewed... Reference is missing. Idem line 19 (It was noted that impairment...).

It was added at P23p3l2 and P23p4l3

11. On page 20, line 22, the authors stated that “One of the possible mechanisms assumed to be involved in impaired memory retrieval in the Y-maze may be a manifest attenuation in synaptic performance during morphine exposure and AMT development”. Indeed, chronic exposure to morphine or heroin leads to the impairment of hippocampal LTP and induces deficits in cognitive and memory-task performance, as shown in the present study. However, the authors right after arguing that “Since morphine may abuse learning and memory circuits, a specific synaptic potentiation, which is the same as LTP, is formed in the brain”. The authors are invited to add a paragraph on this subject to discuss the learning and memory disruption in AMT rats — and the improvement following VPA, as well —, in terms of extinction impairment.

Please look at p22p1-2

12. Graphics are now visually more understandable but progress can be made by applying a title above each figure to avoid the reader going back and forth to the manuscript. Again, I gently ask the authors to increase the size of the fonts since, in case of acceptance of the manuscript, the figures will be reduced in size. Regarding this point, In Figures 1C, 3B, and 3C, the differences between the novel and the other arm, with the start arm, could add a way to easily understand the data. It can just be done by inserting a data inset into the figures.

It was applied in figures1-6 and S1-2

13. Pay attention to several typo errors and others along with the manuscript.

It was done

7. PLOS authors have the option to publish the peer review history of their article ([what does this mean?](https://journals.plos.org/plosone/s/editorial-and-peer-review-process#loc-peer-review-history)). If published, this will include your full peer review and any attached files.


If you choose “no”, your identity will remain anonymous but your review may still be made public.


**Do you want your identity to be public for this peer review?** For information about this choice, including consent withdrawal, please see our [Privacy Policy](https://www.plos.org/privacy-policy).

Reviewer #1: No

[NOTE: If reviewer comments were submitted as an attachment file, they will be attached to this email and accessible via the submission site. Please log into your account, locate the manuscript record, and check for the action link "View Attachments". If this link does not appear, there are no attachment files.]

While revising your submission, please upload your figure files to the Preflight Analysis and Conversion Engine (PACE) digital diagnostic tool, <https://pacev2.apexcovantage.com/>. PACE helps ensure that figures meet PLOS requirements. To use PACE, you must first register as a user. Registration is free. Then, login and navigate to the UPLOAD tab, where you will find detailed instructions on how to use the tool. If you encounter any issues or have any questions when using PACE, please email PLOS at [figures@plos.org](mailto:figures@plos.org). Please note that Supporting Information files do not need this step.
